# Fluconazole Resistance and Heteroresistance in *Cryptococcus* spp.: Mechanisms and Implications

**DOI:** 10.1590/0037-8682-0328-2024

**Published:** 2025-03-24

**Authors:** Izabela de Mesquita Bárcia Moreira, Naira Sulany Oliveira de Sousa, Juan Diego Ribeiro de Almeida, Robert Langlady Lira Rosas, Katia Santana Cruz, Ani Beatriz Jackisch Matsuura, Márcia de Souza Carvalho Melhem, Érica Simplício de Souza, Hagen Frickmann, Marcus Vinícius Guimarães Lacerda, João Vicente Braga de Souza

**Affiliations:** 1 Programa de Pós-Graduação em Biodiversidade e Biotecnologia da Rede BIONORTE, Manaus, AM, Brasil.; 2 Instituto Nacional de Pesquisas da Amazônia, Manaus, AM, Brasil.; 3 Fundação de Medicina Tropical Dr. Heitor Vieira Dourado, Manaus, AM, Brasil.; 4 Instituto Leônidas & Maria Deane, Fiocruz, Manaus, AM, Brasil.; 5 Faculdade de Medicina, PPG Doenças Infecciosas e Parasitárias, UFMS, Campo Grande, MS, Brasil.; 6 Faculdade de Medicina, PPG Doenças Tropicais, Unesp, Botucatu, SP, Brasil.; 7 Instituto de Medicina Tropical de São Paulo, LIM 53, São Paulo, SP, Brasil.; 8 Bundeswehr Hospital Hamburg, Department of Microbiology and Hospital Hygiene, Germany.; 9 University Medicine Rostock, Institute for Medical Microbiology, Virology and Hygiene, Germany.; 10 University of Texas Medical Branch, Galveston, USA.

**Keywords:** *Cryptococcus* spp., heteroresistance, antifungal, fluconazole

## Abstract

The reference methodology for evaluating antifungal susceptibility is based on determining the minimum inhibitory concentration (MIC), which is the lowest drug concentration capable of inhibiting fungal growth. However, such MIC data are insufficient to measure antifungal susceptibility if a strain is heteroresistant to the tested drug. In such cases, a minority subpopulation of fungal cells, originating from an initially susceptible lineage, can grow at antifungal drug concentrations above the MIC. In studies on fluconazole heteroresistance in *Cryptococcus* spp., chromosomal disomy has been shown to result in the overexpression of two genes located on chromosome 1 (Chr1) linked to antifungal resistance: *ERG11* and *AFR1*. This review addresses the underlying mechanisms of antifungal resistance, the evolution of methods for determining antifungal susceptibility, and the clinical implications of *Cryptococcus* heteroresistance to fluconazole. The analysis of the findings indicated a correlation between heteroresistance and adverse clinical outcomes, although this observation still lacks definite confirmation in the literature. This highlights the need to implement more efficient therapeutic strategies and improve antifungal susceptibility and heteroresistance testing.

## INTRODUCTION

Cryptococcosis is a fungal infection caused by *Cryptococcus neoformans* species complex or *Cryptococcus gattii* species complex^1^. Cryptococcal meningitis is the most severe manifestation of this disease. An estimated 152,000 cases per year of cryptococcal meningitis are reported, resulting in 112,000 cryptococcosis-related deaths globally. Cryptococcal disease accounts for 19% of AIDS-related mortality worldwide^2^, while the attributable mortality rates range from 30% to 60% in Latin America^3^. In Brazil, a study from 2000 to 2012 reported a cryptococcosis mortality rate of 619 per million inhabitants, with cryptococcosis as the leading cause of death and 25,2 per million inhabitants with cryptococcosis as an associated cause^4^. In a reference institution in the Brazilian Amazon, cryptococcosis was the sixth leading cause of death^4^. In another reference institution in the Brazilian Amazon, cryptococcosis was the sixth leading cause of death in people living with HIV/AIDS (PLWHA) who underwent autopsy from 1996 to 2003^5^. Isolates of *Cryptococcus* spp. have been shown to tolerate increasingly high concentrations of antifungals, which may explain relapse and therapeutic failure^6^ ^,^ ^7^. Heteroresistance is a phenomenon that consists of the ability of a subpopulation of cells to survive high concentrations of antimicrobially acting drugs like azoles, generating populations of homogeneous cells with high MIC and capable of adapting to even higher concentrations of drugs^8^. 

This review covers the following topics: mechanisms of resistance of *Cryptococcus* spp. against antifungals, evolution of antifungal susceptibility evaluation methods, and clinical implications of heteroresistance, with a particular focus on fluconazole, including studies on the correlation of heteroresistance with clinical outcomes in cryptococcosis patients.

## CRYPTOCOCCOSIS: EPIDEMIOLOGY AND PATHOGENESIS

Cryptococcosis, caused by *Cryptococcus neoformans* and *Cryptococcus gattii*, is a globally distributed fungal disease that causes opportunistic infections in individuals living with HIV/AIDS. Before the advent of highly active antiretroviral therapy, it was a major cause of death among AIDS patients. Despite a global decline in incidence, the disease still has high occurrence and mortality rates in certain regions, including Brazil^9^ ^-^ ^12^. The mortality rate of cryptococcosis is also significantly influenced by the geographical distribution of its etiological agents, with the highest mortality observed in regions with limited access to antiretroviral therapy and adequate healthcare. An African study reported that short-term mortality due to cryptococcal meningitis surpassed 50% in Central and West Africa, compared to 37% in South Africa. This finding confirmed the impact of resource disparities, care intensity, and delays in patient presentation^13^.

Cryptococcosis is caused by the inhalation of *Cryptococcus* spp. propagules, which are phagocytosed by alveolar macrophages, triggering granulomatous inflammation^18^. Persistence within macrophages, formation of a polysaccharide capsule that protects the fungus from adverse conditions, such as phagocytosis next to other antiphagocytic mechanisms, production of melanin and degradative enzymes, and the ability of living at environmental temperatures, are examples of these mechanisms^16^. The severity and dissemination of the disease are determined by the patient's immune status, with meningoencephalitis being the most common manifestation in immunocompromised patients. *Cryptococcus neoformans* genotypes VNI, VNII, and VNIII are found globally, whereas VNIV is mainly reported in Europe*. Cryptococcus gattii* VGII typically inhabits tropical and subtropical regions; however, in 1999, an outbreak occurred in the non-tropical areas of Canada and northwestern USA^14^. Studies indicate that *C. gattii* in general, and genotype VGII in particular, which is considered highly virulent, originated from the North and Northeast of Brazil^15^.

## RESISTANCE OF CRYPTOCOCCI AGENTS TO FLUCONAZOLE

The medical treatment for cryptococcosis consists of three sequential phases: induction, consolidation, and maintenance. The induction phase aims at rapidly reducing the fungal burden, typically with intravenous liposomal amphotericin B at 3 mg/kg/day combined with oral flucytosine at 100 mg/kg/day, divided into four daily doses administered for at least two weeks. The consolidation phase is added to sustain the initial therapeutic response and prevent relapse, which can be achieved with oral fluconazole at 400-800 mg/day for a minimum of eight weeks. Finally, the maintenance phase focuses on preventing recurrence, particularly in immunocompromised patients, by administering oral fluconazole at 200 mg/day for a minimum of 12 months. This protocol, as recommended by the Brazilian Ministry of Health, is similar to treatment guidelines in other parts of the world^16^ ^-^ ^29^. Notably, fluconazole can be applied at various stages, which motivates the discussion in this review article.

**FIGURE 1:**
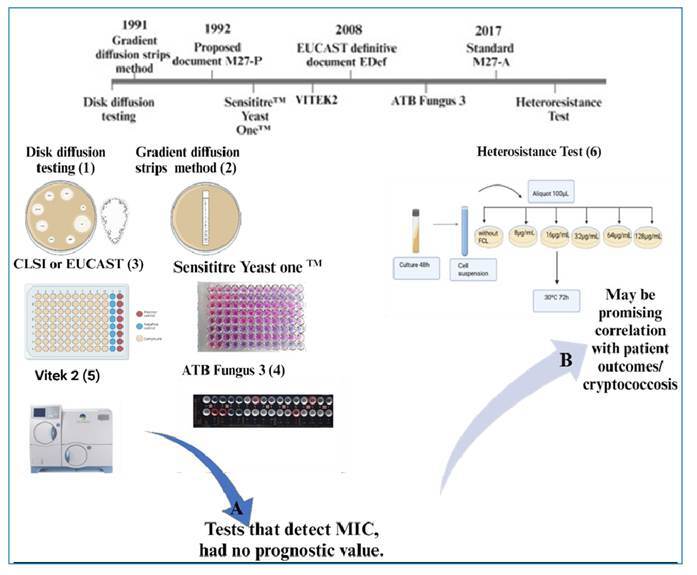
Historical evolution of antifungal susceptibility assessment methods. **(A)** Conventional methods currently used for Antifungal Susceptibility Testing (AFST) that determine MIC have no prognostic value. **(B)** An approach correlating in vivo data and in vivo response may be a promising in indicating clinical outcomes **(1,2)**. **(1)** Disk diffusion testing. **(2)** Gradient strips diffusion method (epsilon-diffusion testing); **(3)** Broth microdilution method in a 96 well microplate: EUCAST and CLSI references and Sensititre Yeast One (YstO™); **(4)** ATB Fungus 3(ATB) ^TM^. **(5)** Automated Antifungal Susceptibility System - Vitek 2^TMS^ (Vitek); **(6)** Heteroresistance testing.

Growing concerns over antifungal resistance, especially in cases such as cryptococcosis, have led to the development and standardization of *in vitro* susceptibility testing methods. The disk diffusion test is an initial technique that uses paper disks with fixed antifungal concentrations. Gradient diffusion strips for epsilon diffusion tests later became a common alternative, providing the advantage of determining MIC values^30^. In the 1980s, the Clinical and Laboratory Standards Institute (CLSI) began standardizing these tests, resulting in the M-27 document in 1997. The European Committee on Antimicrobial Susceptibility Testing (EUCAST) also formed a subcommittee on antifungal testing and issued recommendations in 2008^31^. Current CLSI standards are outlined in M27-A4, with rapid automated methods like Vitek 2™ and SensititreYeast One™ using broth microdilution^32^. Figure 1 summarizes the historical evolution of antifungal susceptibility tests, their relationship with clinical prognosis, and the need for new tests to detect heteroresistance phenomena^6^ ^,^ ^7^ ^,^ ^33^ ^,^ ^34^. 

According to the M27 A4 standard, the minimum inhibitory concentration (MIC) of fluconazole is defined as the concentration that reduces fungal growth by 50% compared with the control^35^. Breakpoints (BP) for *Cryptococcus* species have not been established and thus, breakpoint values used for *Candida* spp. are often extrapolated: MIC ≤ 8 µg/mL as susceptible, 16-32 µg/mL as dose-dependent susceptible, and >64 µg/mL as resistant. Because the CLSI does not provide BPs for *Cryptococcus* spp., only epidemiological cutoff values (ECOFFs) are available to classify isolates as non-wild-type without directly indicating resistance or susceptibility^24^ ^,^ ^36^. An international study proposed ECOFF values for the *C. neoformans* species complex, genotype VNI, as 8 µg/mL, for the genotypes VNIII and VNIV as 16 µg/mL. For the *C. gattii* species complex genotypes VGI, VGIIa and VGIII, the proposed ECOFF was 8 µg/mL, for the genotypes VGII and VGIV, it was 32 µg/mL and 16 µg/mL, respectively^36^ ^,^ ^37^.

However, previous studies have demonstrated that diagnostic antifungal susceptibility testing cannot uniformly predict treatment success or failure. In particular, a high MIC value, quantified according to the CLSI or EUCAST, is not necessarily associated with increased patient mortality^30^. This challenge is particularly evident in the treatment of cryptococcosis, a condition associated with high mortality rates and frequent therapeutic failures^38^ ^-^ ^41^. The correlation between *in vitro* fluconazole data and clinical response is not always well-defined^42^ ^,^ ^43^. More than 4% of 143 AIDS patients experienced relapse during extended fluconazole therapy, with fluconazole MICs increasing 8- to 12-fold in serial isolates over up to 5 months^44^. Fluconazole treatment failure in *C. neoformans* infections was linked to *in vitro* MIC data, showing values ≥64 mg/L^45^. 

**FIGURE 2:**
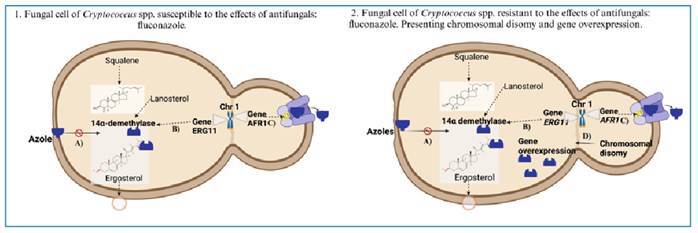
Schematic presentation of susceptible **(1)** and resistant **(2)** *Cryptococcus* spp. cells and the mechanisms of action of azoles in fungal cells, next to molecular mechanisms of resistance of *Cryptococcus* spp. against fluconazole. Ergosterol from the fungal membrane of *Cryptococcus* spp. is formed from squalene mediated by the enzyme 14-α demethylase. Fluconazole, from the azoles class, acts by inhibiting the enzyme 14-α demethylase **(A)**. The genes *ERG11*, which encodes the enzyme 14-α demethylase (33)**(B)**, and *AFR1*, which is responsible for the efflux of azoles via an ATP pump(51), are shown **(C)**. As a way of adapting to high concentrations of antifungals, chromosomal disomy occurs, resulting in overexpression of two genes located on chromosome 1 (Chr1), *ERG11* and *AFR1* **(D)**. Created with BioRender.com

The cause of fluconazole resistance in *Cryptococcus* spp. is not yet fully understood^24^ ^,^ ^33^. Figure 2 shows a schematic representation of the mechanism of action of azoles on fungal cells and potential resistance” of *Cryptococcus* spp. to fluconazole.

Previous studies on *Cryptococcus* spp. *in-vitro* FLC resistance indicated relevance of chromosomal disomy resulting in overexpression of the *ERG11* gene, which encodes the fluconazole target enzyme lanosterol 14α-demethylase, or improved azole efflux mediated by the *AFR1* gene, which encodes the ATP-binding cassette transporter facilitating azole efflux^47^ ^-^ ^49^. The two genes *ERG11* and *AFR1* are located on chromosome 1 (Chr1), which is primarily duplicated as an adaptation to increasing azole concentration^33^. Figure 2 also shows that the enzyme lanosterol 14α-demethylase is crucial for forming the fungal membrane element ergosterol from squalene. It is a target enzyme of azoles that inhibit fungal ergosterol formation.

Academic debate focuses on the question whether “resistance mechanisms” or “heteroresistance mechanisms” are observed when elevated fluconazole MICs for *Cryptococcus* spp. are reported. Several mechanisms presently attributed to antifungal resistance, including those indicated in Figure 2, are known to show only transient effects. In fact, MIC values decreased when *Cryptococcus* spp. were removed from the FLC exposure. Considering traditional definitions of resistance as a stable mechanism, it is essential to discuss whether some of the high MIC values reported in literature may just indicate transient changes associated with heteroresistance rather than “traditional” resistance^33^ ^,^ ^50^ ^,^ ^51^.

## HETERORESISTANCE AGAINST FLUCONAZOLE: CONCEPT AND MECHANISMS

**FIGURE 3:**
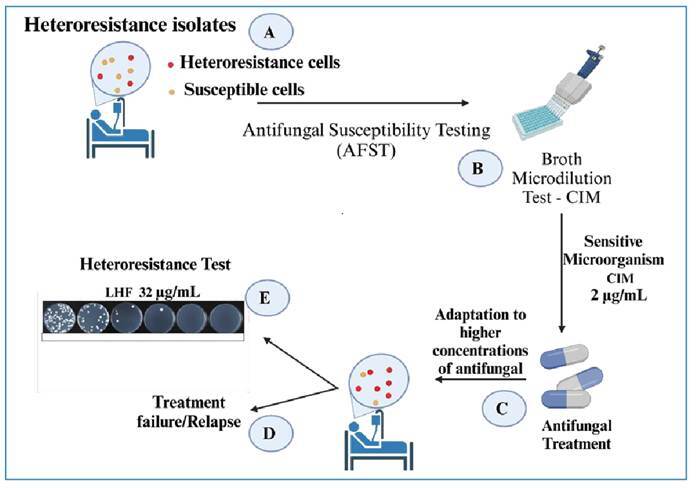
Heteroresistance and antifungal susceptibility testing. **(A)** A patient infected with *Cryptococcus* spp., for which the microbial population appears homogeneous, but some cells can withstand high concentrations of antifungal agents. **(B)** The antifungal susceptibility test, using the microdilution method, with an MIC of 2 ug/mL indicates susceptibility to fluconazole. In this conventional test, the heteroresistant subpopulation is not detected. **(C)** Antifungal treatment based on the results of antifungal susceptibility testing. During treatment, heteroresistant microorganisms adapt to increasingly higher concentrations of antifungal agents. **(D)** Inadequate antifungal therapy leads to treatment failure or relapses. **(E)** Heteroresistance tests are not yet used in clinical practice; however, they can more reliably detect the maximum concentration that these microorganisms can withstand. In this example, the level of fluconazole heteroresistance (LHF) reached 32 ug/mL. This test can help adjusting antifungal treatment and potentially achieving a more favorable clinical outcome.

Azole heteroresistance occurs in a resistant subpopulation of susceptible strains. These cells are capable of surviving high concentrations of azoles, resulting in a homogeneous population with elevated MICs that can adapt to even higher drug concentrations, whereas the progenitor cells from which they originate remain susceptible. This phenomenon is generally unstable; once the selective pressure of the drug is removed, heteroresistant cells may revert to their original susceptible phenotype^46^ ^,^ ^52^. Heteroresistance is a transient phenomenon that relies on specific conditions such as the presence of antimicrobial agents. In the *Cryptococcus neoformans* complex, fluconazole heteroresistance is an intrinsic characteristic associated with virulence, although no correlation has been observed with the molecular type of *Cryptococcus* spp.^6^ ^,^ ^7^ ^,^ ^34^. Figure 3 demonstrates the heteroresistance phenomenon and shows what occurs during susceptibility testing.

Since 1947, heteroresistance has been observed in both Gram-positive and Gram-negative bacteria^53^. This has been increasingly observed across various microbial pathogens, allowing a subset of cells within a population to survive antibiotic exposure, while the majority of cells remain susceptible. Notably, this phenomenon has been studied in bacterial species such as *Escherichia coli, Klebsiella pneumoniae*, and *Acinetobacter baumannii*, particularly in response to antibiotics such as polymyxins, carbapenems, and aminoglycosides^54^ ^-^ ^56^. Among fungi, *Nakaseomyces glabratus (*formerly called *Candida glabrata)* can develop heteroresistance to antifungal agents, such as fluconazole, primarily through transient mechanisms, such as the upregulation of efflux pumps, which allows subsets of cells to temporarily withstand drug exposure^50^. This phenomenon may be closely related to antifungal therapeutic failure because higher drug resistance is observed in isolates recovered from infected individuals when the disease recurs.

Unfortunately, the available methods for evaluating FLC heteroresistance have not yet been standardized. A population analysis profile (PAP) assay was used to evaluate the heteroresistance of several microorganisms, including *Cryptococcus* spp. This method measures the ability of cell subpopulations to survive at antifungal concentrations higher than their MIC. For this assay, a defined suspension of fungal cells is cultured on agar plates containing different concentrations of fluconazole. After the incubation period, the colonies growing at each concentration are counted, resulting in a profile which revealed the presence of heteroresistant subpopulations^34^ ^,^ ^46^. According to this definition, the level of FLC heteroresistance is the maximum FLC concentration that allows the growth of a subpopulation of an isolate. FLC resistance-induction assays have also been proposed^7^ ^,^ ^34^ ^,^ ^46^. In these assays, isolates are subjected to a gradual increase of the antifungal drug dose to induce the survival of individual cells at the maximum possible concentration of the inhibitory substance^7^ ^,^ ^46^. In the case of heteroresistance stability assessment, the test result is the number of passages in the drug-free medium required to return to the initial MIC^34^ ^.^

The process of heteroresistance induction in strains of *Cryptococcus neoformans* against fluconazole was described by Mondon et al. (1999)^52^. During a recent evaluation of 107 clinical isolates of *C. neoformans*, four were capable of growing in a medium containing fluconazole at concentrations four to eight times higher than the MICs originally determined for these specific isolates^57^. The level of FLC heteroresistance in 100 clinical and environmental isolates of *Cryptococcus* spp. was investigated in southeastern region of Brazil^7^. A Brazilian study investigated fluconazole heteroresistance in clinical and environmental isolates of *Cryptococcus neoformans* and *Cryptococcus gattii* complexes from Amazonas, Brazil. This study revealed heteroresistance in several isolates^6^. Isolates with high LHF were significantly more virulent than those with low levels. The respective study also highlighted a significantly higher proportion of *Cryptococcus gattii* isolates (86%) compared to *Cryptococcus neoformans* (46%) that exhibited LHF levels ≥16 µg/mL^34^. 

Various studies have demonstrated that heteroresistance in *Cryptococcus neoformans* is characterized by transient chromosomal disorders in response to fluconazole-induced stress^58^. For instance, duplication of chromosome 1, which harbors crucial genes such as *ERG11* (fluconazole target) and *AFR1* (ABC transporter), was observed both *in vitro* and in the brains of fluconazole-treated mice^33^. Chr4 was the second most frequent chromosome, with a disomic occurrence at high fluconazole concentrations^59^. A 2010 study using genome hybridization and real-time PCR showed that *Cryptococcus neoformans* adapted to high fluconazole concentrations through chromosomal duplication^60^. Stone et al. demonstrated heteroresistance in *Cryptococcus* spp. both *in vitro* and *ex vivo*. They used a PAP assay to show that aneuploidy, such as chromosomal duplication, is a key element of heteroresistance. Using a hollow fiber infection model and a murine model, they linked fluconazole exposure to the selection of resistant subpopulations and identified chromosome 1 duplication as the major mechanism^8^.

In summary, the current studies emphasize the significance of heteroresistance in *Cryptococcus* spp., particularly to fluconazole, a widely used antifungal agent. Although mechanisms, such as transient chromosomal disorders, are well documented, there are notable limitations regarding the evaluation methods and clinical applicability of these findings. The lack of standardized tests for heteroresistance and the complexity of fungal adaptation under antifungal pressure make it challenging to accurately predict therapeutic failures. Future perspectives include the development of more robust diagnostic methods to detect and monitor heteroresistance along with ongoing research on new treatments and strategies to manage resistant subpopulations and improve clinical outcomes. 

## HETERORESISTANCE AGAINST FLUCONAZOLE AND CLINICAL OUTCOMES IN CRYPTOCOCCOSIS

The relationship between antifungal resistance mechanisms and clinical outcomes in cryptococcosis remains a subject of ongoing debate. Although MIC and heteroresistance determined by PAP have been investigated as predictors of clinical outcomes, there is limited evidence supporting a direct link between these metrics and patient responses or mortality rates.

The hypothesis that "high levels of heteroresistance" correlate with "high mortality" implies that isolates with increased heteroresistance can withstand higher drug concentrations, potentially leading to persistent infection and treatment failure^8^ ^,^ ^61^. However, the translation of *in vitro* resistance metrics into *in vivo* outcomes is complex. This complexity is due to factors such as the patient’s immune status, variability in the metabolism and distribution of antifungal drugs in the human body, and differences in the fungal burden and virulence. 

Few studies have analyzed the clinical response to treatment and mortality rates in relation to the *in vitro* resistance characteristics of isolates, such as MIC values and heteroresistance levels. In addition, a study showed that animals infected with high-level heteroresistant clones experienced a significant increase in mortality (80-90%) compared to animals infected with low-level heteroresistant cells (0-10% mortality) over a short period of time^46^. In 2019, Stone et al. demonstrated that dynamic changes in ploidy contribute to fluconazole resistance in human cryptococcal meningitis, highlighting a reversible and adaptive mechanism that enables *Cryptococcus* spp. to survive antifungal pressure^8^. A study by de Oliveira et al. identified high fungal burden in the cerebrospinal fluid, low CD4+ T-lymphocyte count, and elevated inflammatory protein levels at the start of treatment as more significant indicators of poor prognosis than fluconazole heterosistance^41^. Even in larger study populations, as assessed in 2023, death-related variables are not convincingly associated with heteroresistance^62^.

We conclude that current studies on fluconazole heteroresistance in cryptococcosis are limited by the challenges in translating *in vitro* data into clinical outcomes. Factors, such as the patient's immune status, drug pharmacokinetics, and fungal virulence, complicate clear correlations. Obstacles like small sample size and lack of standardized tests make definitive conclusions difficult. Future research should include larger standardized longitudinal clinical studies to confirm the association between clinical impact and heteroresistance. 

## CONCLUSIONS

- This review elucidates the complex mechanisms underlying antifungal resistance in *Cryptococcus* spp., including transient chromosomal disorders and efflux pumps that facilitate fungal survival under fluconazole exposure. These adaptive responses suggest heteroresistance, with fungal cells maintaining the ability to revert to susceptibility after the antifungal drug pressure is removed, which is in contrast to traditional definitions of stable resistance.
- The evolution of antifungal susceptibility testing has provided critical insights into *Cryptococcus* spp. responses to fluconazole. However, current methods lack the sensitivity required to consistently detect heteroresistance. Standardized approaches, such as the CLSI and EUCAST protocols, provide increased diagnostic accuracy, but are limited in their ability to predict clinical outcomes. New methodologies such as population analysis profiling (PAP), are required to identify heteroresistance levels more accurately.
- Evaluating the clinical relevance of heteroresistance in *Cryptococcus* spp. provides complex challenges. In particular, high FLC heteroresistance levels correlate with increased virulence. However, translating *in vitro* heteroresistance metrics into definitive clinical outcome predictions is complicated by individual variations in immune status and drug metabolism. Future studies should focus on standardizing heteroresistance testing and conducting large-scale longitudinal studies to clarify the impact of heteroresistance on prognosis and treatment success.
